# Vesicular glutamate transporter modulates sex differences in dopamine neuron vulnerability to age‐related neurodegeneration

**DOI:** 10.1111/acel.13365

**Published:** 2021-04-28

**Authors:** Silas A. Buck, Thomas Steinkellner, Despoina Aslanoglou, Michael Villeneuve, Sai H. Bhatte, Victoria C. Childers, Sophie A. Rubin, Briana R. De Miranda, Emma I. O'Leary, Elizabeth G. Neureiter, Keri J. Fogle, Michael J. Palladino, Ryan W. Logan, Jill R. Glausier, Kenneth N. Fish, David A. Lewis, J. Timothy Greenamyre, Brian D. McCabe, Claire E. J. Cheetham, Thomas S. Hnasko, Zachary Freyberg

**Affiliations:** ^1^ Center for Neuroscience University of Pittsburgh Pittsburgh PA USA; ^2^ Department of Psychiatry University of Pittsburgh Pittsburgh PA USA; ^3^ Department of Neurosciences University of California, San Diego La Jolla CA USA; ^4^ Institute of Pharmacology Center for Physiology and Pharmacology Medical University of Vienna Vienna Austria; ^5^ Department of Neurology University of Pittsburgh Pittsburgh PA USA; ^6^ Department of Pharmacology & Chemical Biology University of Pittsburgh Pittsburgh PA USA; ^7^ Pittsburgh Institute for Neurodegenerative Diseases University of Pittsburgh Pittsburgh PA USA; ^8^ Department of Pharmacology and Experimental Therapeutics Boston University School of Medicine Boston MA USA; ^9^ Center for Systems Neurogenetics of Addiction The Jackson Laboratory Bar Harbor ME USA; ^10^ Geriatric Research, Education and Clinical Center VA Pittsburgh Healthcare System Pittsburgh PA USA; ^11^ Brain Mind Institute Swiss Federal Institute of Technology (EPFL) Lausanne Switzerland; ^12^ Department of Neurobiology University of Pittsburgh Pittsburgh PA USA; ^13^ Research Service VA San Diego Healthcare System San Diego CA USA; ^14^ Department of Cell Biology University of Pittsburgh Pittsburgh PA USA; ^15^ Present address: Department of Neurology Center for Neurodegeneration and Experimental Therapeutics University of Alabama at Birmingham Birmingham AL USA

**Keywords:** aging, dopamine, *Drosophila*, neurodegeneration, vesicular glutamate transporter

## Abstract

Age is the greatest risk factor for Parkinson's disease (PD) which causes progressive loss of dopamine (DA) neurons, with males at greater risk than females. Intriguingly, some DA neurons are more resilient to degeneration than others. Increasing evidence suggests that vesicular glutamate transporter (VGLUT) expression in DA neurons plays a role in this selective vulnerability. We investigated the role of DA neuron VGLUT in sex‐ and age‐related differences in DA neuron vulnerability using the genetically tractable *Drosophila* model. We found sex differences in age‐related DA neurodegeneration and its associated locomotor behavior, where males exhibit significantly greater decreases in both DA neuron number and locomotion during aging compared with females. We discovered that dynamic changes in DA neuron VGLUT expression mediate these age‐ and sex‐related differences, as a potential compensatory mechanism for diminished DA neurotransmission during aging. Importantly, female *Drosophila* possess higher levels of VGLUT expression in DA neurons compared with males, and this finding is conserved across flies, rodents, and humans. Moreover, we showed that diminishing VGLUT expression in DA neurons eliminates females' greater resilience to DA neuron loss across aging. This offers a new mechanism for sex differences in selective DA neuron vulnerability to age‐related DA neurodegeneration. Finally, in mice, we showed that the ability of DA neurons to achieve optimal control over VGLUT expression is essential for DA neuron survival. These findings lay the groundwork for the manipulation of DA neuron VGLUT expression as a novel therapeutic strategy to boost DA neuron resilience to age‐ and PD‐related neurodegeneration.

## Graphical abstract

Dopamine neuron VGLUT mediates age‐ and sex‐related differences in dopamine neurodegeneration during aging. Female *Drosophila*, rats and humans express more VGLUT in dopamine neurons than males, and VGLUT knockdown in dopamine neurons diminishes sex differences in vulnerability to age‐related degeneration. The ability to finely tune dopamine neuron VGLUT expression boosts resilience while either too little or too much VGLUT leaves dopamine neurons more vulnerable to neurodegeneration.

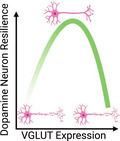

## 1 INTRODUCTION

Progressive loss of midbrain dopamine (DA) neurons and their striatal projections are defining features of Parkinson's disease (PD) (Hartmann, 2004). Aging is the greatest risk factor for PD (Reeve et al., 2014), yet little is known about the mechanisms that determine DA neuron vulnerability across age. Not all DA neurons are equally susceptible to neurodegeneration (Giguere et al., 2018), as DA neurons in the ventral tegmental area (VTA) are more resilient than DA neurons of the substantia nigra *pars compacta* (SNc) (Sulzer & Surmeier, 2013). The capacity of DA neurons for compensatory plasticity may shed new light on the mechanisms underlying selective DA neuron vulnerability (Brotchie & Fitzer‐Attas, 2009) including why individuals can lose ~80% of their SNc DA neurons and remain asymptomatic for the motor symptoms of PD (Brotchie & Fitzer‐Attas, 2009).

The more resilient VTA DA neurons have unique physiological properties that offer key clues for understanding the mechanisms of selective vulnerability to age‐related DA neurodegeneration. Medial VTA DA neurons fire at significantly higher frequencies (>20 Hz) for extended periods compared with other midbrain dopaminergic populations (Chuhma et al., 2009; Grace & Bunney, 1984). Such firing rates require the cells to employ an adaptive mechanism for tuning neurotransmission in response to prolonged elevated activity. Notably, a higher proportion of these VTA DA neurons co‐release glutamate and express vesicular glutamate transporter 2 (VGLUT2) which is responsible for the vesicular packaging of glutamate (Mingote et al., 2019; Trudeau & El Mestikawy, 2018). We previously showed that VGLUT2 can also enhance vesicle loading and release of DA in response to increased neuronal activity (Aguilar et al., 2017). However, the precise role of VGLUT2 expression in DA neuron resilience remains controversial. Adult DA neurons can upregulate VGLUT2 in response to cell stress as an adaptive, neuroprotective response to insult (Kouwenhoven et al., 2020; Shen et al., 2018; Steinkellner et al., 2018). Nevertheless, other data suggest that excess VGLUT2 expression can induce DA neurodegeneration (Steinkellner et al., 2018). Moreover, while females have lower prevalence of PD, the molecular mechanisms underlying this sex difference remain unknown.

To address these outstanding questions, we employed the genetically tractable *Drosophila* model. We show that flies exhibit age‐related DA neuron degeneration similar to mammals, with females less vulnerable than males. Further, we found that DA neuron VGLUT expression is dynamically upregulated during aging and preventing this age‐related upregulation of VGLUT in DA neurons eliminates sex differences in DA neuron degeneration during aging. Significantly, we discovered that the sex differences in DA neuron VGLUT expression are conserved across species from flies to rodents to humans. Finally, we translate our findings to mammals, demonstrating in mice that the delicate balance of VGLUT expression in DA neurons is critical for its effects on resilience. Overall, our data suggest that VGLUT's sex differences in DA neurons are highly conserved and that the regulatory mechanisms that control the balance of VGLUT2 expression in DA neurons may play an important role in selective DA neuron vulnerability and underlie female resilience to age‐related DA neuron degeneration.

## 2 RESULTS

### 2.1 Aging causes sex‐specific locomotor loss in *Drosophila* coupled with dVGLUT upregulation

**FIGURE 1:**
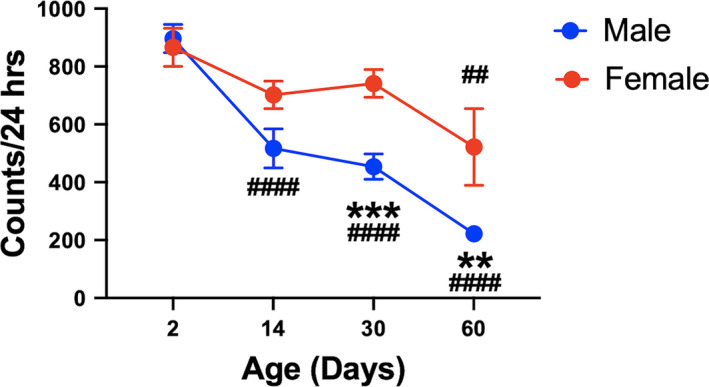
Sex differences in age‐dependent locomotor loss in *Drosophila*. Adult male flies show progressive loss of 24‐h locomotion across aging compared with females who only show diminished locomotion at day 60 post‐eclosion. Results represented as mean ± SEM; **p < 0.01, ***p < 0.001 compared with opposite sex, ^##^ *p* < 0.01, ^####^ *p* < 0.0001 compared with locomotion in 2‐day‐old flies by Bonferroni post hoc comparison; *n* = 11–31 animals per group

As in mammals, DA neurons play a critical role in locomotion in *Drosophila* (Bainton et al., 2000; Yamamoto & Seto, 2014), but it is unclear whether flies also exhibit similar age‐related and sex‐specific motor differences. We therefore monitored 24‐h basal locomotion of male and female wild‐type w^1118^ flies across the lifespan (Figure 1). We found a significant loss in basal locomotion as a function of aging (Age effect: *F*_3,169_ = 26.9, *p* < 0.0001) and discovered that this age‐related locomotor decline was greater in males than females (Sex effect: *F*_1,169_ = 20.7, *p* < 0.0001, Age × Sex interaction: *F*_3,169_ = 4.1, *p* = 0.0072) (Figure 1). Consistent with mammals, these findings reveal greater susceptibility to age‐dependent loss of locomotion in male flies.

**FIGURE 2:**
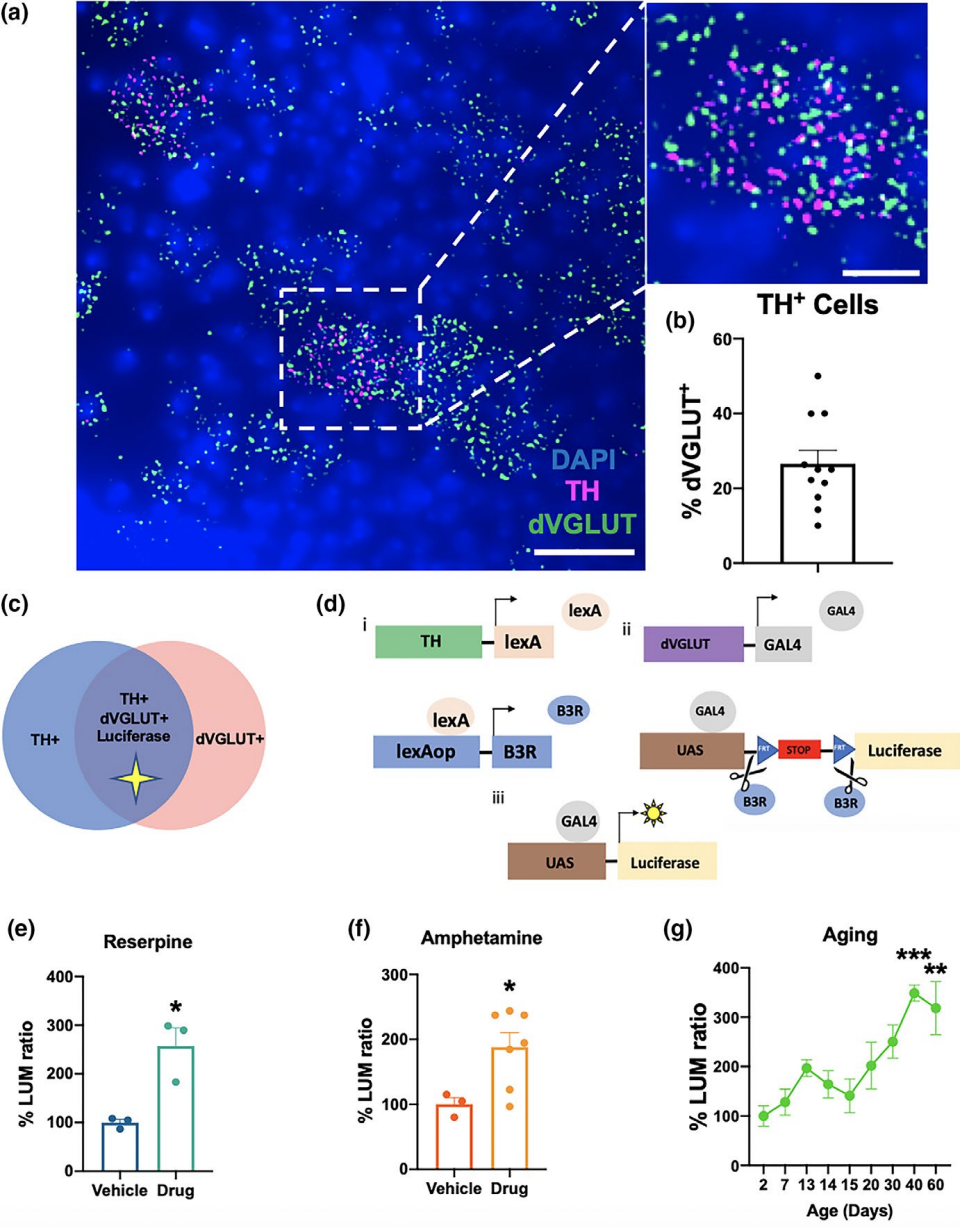
Intersectional genetic reporter shows altered DA neuron dVGLUT expression in response to vesicular DA depletion and to sex. (a) Representative confocal image demonstrating TH (magenta) and dVGLUT (green) mRNA expression via multiplex RNAscope in neurons of wild‐type w^1118^ fly central brain 14 days post‐eclosion; scale bar = 25 μm. Inset shows a zoomed‐in cell expressing both TH and dVGLUT mRNA; scale bar = 10 μm. (b) Quantification of the fraction of TH^+^ neurons that express dVGLUT via RNAscope labeling in adult central brain shows 27 ± 3.7% of TH^+^ DA neurons are also dVGLUT^+^ (*n* = 11 brains). (c) Schematic where an intersectional genetic luciferase reporter of dVGLUT expression is expressed only in DA neurons that express both TH and dVGLUT. (d) Panel i: The TH promoter drives LexA to express B3 recombinase (B3R) in TH^+^ cells. Panel ii: B3R excises a transcriptional stop cassette within UAS‐Luciferase. Panel iii: This permits successful dVGLUT‐GAL4‐driven transactivation of UAS‐Luciferase selectively in TH^+^/dVGLUT^+^ cells. (e) Vesicular DA depletion by reserpine (300 μM, 24 h) significantly upregulates dVGLUT expression in DA neurons compared with vehicle (2.6‐fold increase; *t*_4_ = 3.8, *p* = 0.020). (f) Amphetamine (10 mM, 24 h) also increases DA neuron dVGLUT expression 1.9‐fold versus vehicle (*t*_8_ = 2.5, *p* = 0.039). (g) Increasing age progressively increases DA neuron dVGLUT expression (one‐way ANOVA: *p* < 0.0001) with a 3.5‐fold increase in dVGLUT expression by day 40 versus day 2 post‐eclosion (Bonferroni post hoc test: *p* = 0.0001). Results represented as mean ± SEM; *p < 0.01 versus vehicle, **p < 0.01, ***p < 0.001 versus Day 2; *n* = 3–7 homogenates of 5 brains per group in intersectional luciferase reporter studies

We and others have shown that mammalian DA neurons alter expression of VGLUT2 in response to cell stress, emphasizing VGLUT's potential importance in mechanisms of DA neuron plasticity and selective vulnerability (Kouwenhoven et al., 2020; Shen et al., 2018; Steinkellner et al., 2018). We thus investigated whether DA neuron‐specific changes in VGLUT expression may be critical in age‐related DA neurodegeneration in our fly model. First, in situ hybridization via multiplex RNAscope confirmed the presence of tyrosine hydroxylase (TH)‐expressing DA neurons that also express *Drosophila* VGLUT (dVGLUT) in the central brain of adult wild‐type w^1118^ flies (Figure 2a). We identified 27 ± 3.7% of TH^+^ DA neurons are concurrently dVGLUT^+^ (Figure 2b), similar to estimates of TH^+^/VGLUT2^+^ neurons in rodent midbrain (Mingote et al., 2017; Yamaguchi et al., 2007). We then employed a recombinase‐induced intersectional genetic method to express a reporter of dVGLUT expression specifically in TH^+^/dVGLUT^+^ DA neurons (Figure 2c). Since TH is the rate‐limiting enzyme of DA biosynthesis, we constructed a fly strain using the TH‐LexA expression driver, where B3 recombinase (B3R) is targeted to TH^+^ DA neurons to excise a stop cassette. This permits expression of a firefly luciferase transcriptional reporter driven by the dVGLUT promoter only in TH^+^ DA neurons (Figure 2d). Consequently, our intersectional luciferase reporter produces a robust DA neuron‐specific luminescent indicator of dVGLUT expression level with high signal‐to‐noise compared with controls (Figure S1). Expression of the DA neuron dVGLUT reporter is consistent with previous work in *Drosophila* demonstrating dVGLUT protein in a subset of DAergic nerve terminals (Aguilar et al., 2017).

With our luciferase reporter, we first examined whether DA neurons dynamically alter dVGLUT expression following depletion of vesicular DA. We used reserpine (300 μM, 24 h), which depletes vesicular DA stores by blocking the vesicular monoamine transporter (VMAT) to prevent loading of cytoplasmic DA into the vesicle lumen, leading to DA degradation (Varoqui & Erickson, 1997). Reserpine treatment increases reporter output 2.6‐fold compared with vehicle (unpaired *t* test: *t*_4_ = 3.8, *p* = 0.020) (Figure 2e). We next tested amphetamine (10 mM, 24 h), which also depletes presynaptic DA stores, albeit via a mechanism different from reserpine. Amphetamine disrupts the vesicular pH gradient (ΔpH), the main driving force for vesicular DA loading and retention. This leads to movement of DA out of the vesicle into the cytoplasm where it is transported out of the neurons via amphetamine‐induced dopamine transporter efflux, resulting in DA depletion (Freyberg et al., 2016). Similar to reserpine, amphetamine increases luciferase reporter expression 1.9‐fold (*t*_8_ = 2.5, *p* = 0.039) (Figure 2f). Next, in the context of aging, we observed a threefold increase in luciferase reporter expression in older adult flies aged 40‐ (Bonferroni post hoc test: *p* = 0.0001) and 60 days (*p* = 0.0010) post‐eclosion compared with young flies (day 2 post‐eclosion) (Figure 2g), suggesting a maximum level of dVGLUT upregulation is reached in DA neurons by 40 days. Together, our findings show that dVGLUT expression in DA neurons is highly dynamic, potentially as a compensatory response to changes in synaptic DA levels.

### 2.2 Sex differences in DA neuron VGLUT expression are conserved across species

**FIGURE 3:**
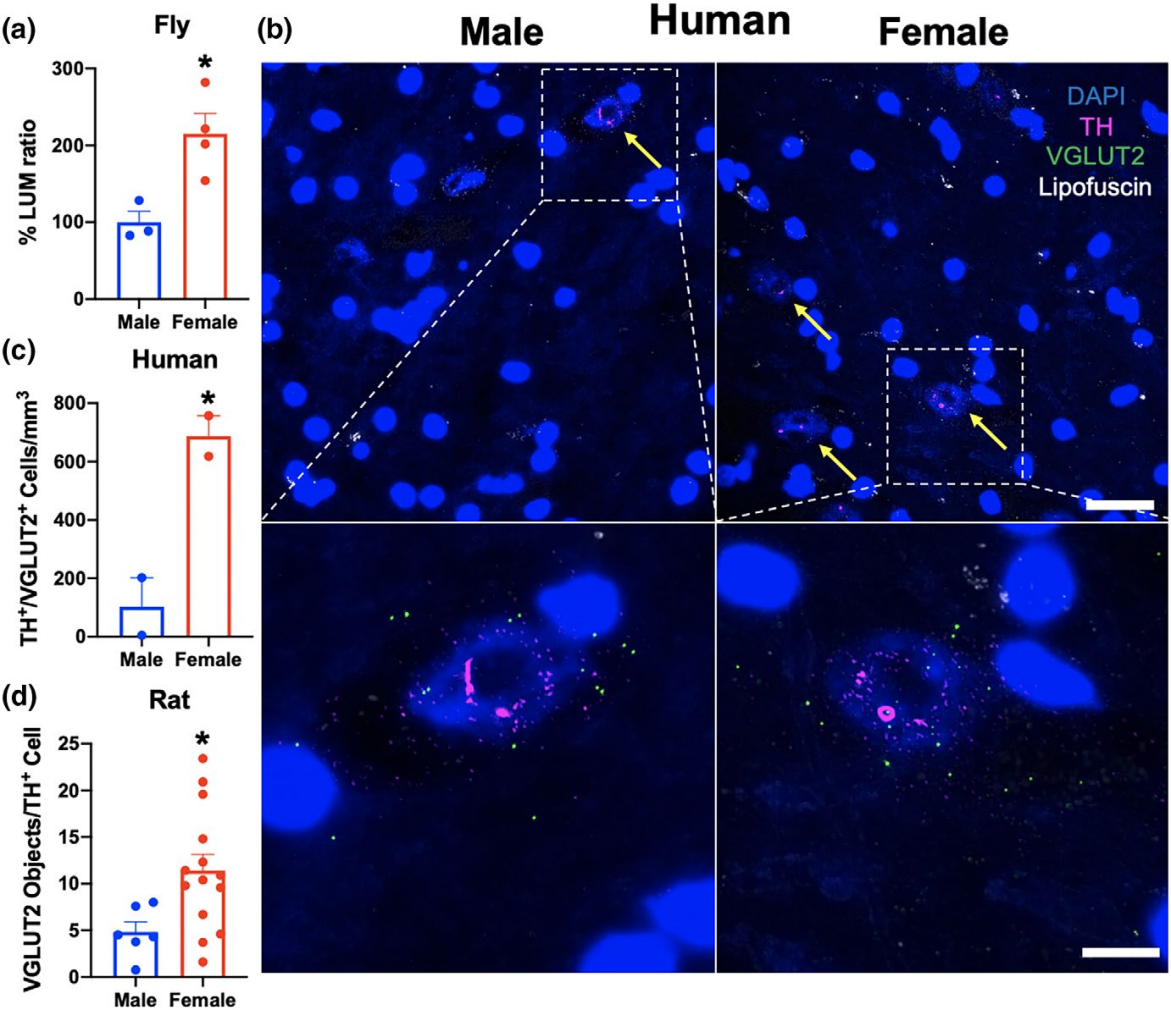
Sex differences in DA neuron VGLUT expression. (a) Using DA neuron dVGLUT luciferase reporter flies, we observed sex differences in DA neuron dVGLUT expression with female flies expressing 2.1‐fold more dVGLUT compared with males at 14 days post‐eclosion (*t*_5_ = 3.4, *p* = 0.019); *n* = 3–4 homogenates of five brains per group. (b) In postmortem human VTA/SNc of male and female subjects, representative images of multiplex fluorescent in situ hybridization demonstrate TH (magenta) and VGLUT2 (green) mRNA expression; yellow arrows denote TH^+^/VGLUT2^+^ neurons. Whole field image scale bar = 25 μm, inset scale bar = 10 μm. (c) Human TH^+^/VGLUT2^+^ neuron density is 6.6 times higher in female subjects compared with males (*t*_2_ = 4.8, *p* = 0.040); *n* = 2 subjects per group. (d) Rat TH^+^ midbrain neurons show 2.4‐fold elevated VGLUT2 protein expression in females versus males via immunohistochemistry (*t*_18_ = 2.4, *p* = 0.029); *n* = 6 males, 14 females. Results represented as mean ± SEM; *p < 0.05 by unpaired *t* test

Since female flies show diminished vulnerability to age‐related locomotor decline relative to males, and DA neurons upregulate dVGLUT during aging, we examined whether there are sex differences in DA neuron dVGLUT expression using our intersectional luciferase reporter flies. We discovered that sex differences in DA neuron dVGLUT expression are dynamic across aging and that the most pronounced expression differences between adult males and females occur at day 14 post‐eclosion (data not shown), providing a rationale for focusing on this timepoint. Thus, on day 14 post‐eclosion, adult females express 2.1‐fold more dVGLUT in DA neurons compared with males (*t*_5_ = 3.4, *p* = 0.019) (Figure 3a). We also asked whether these differences are conserved across species. Using multiplex fluorescent in situ hybridization of TH and VGLUT2, we found that the relative density of TH^+^/VGLUT2^+^ neurons in human VTA/SNc is 6.6 times greater in females than in males (*t*_2_ = 4.8, *p* = 0.040) (Figure 3b,c, Table S1). Furthermore, adult female rats similarly express 2.4‐fold more VGLUT2 protein in DA neurons versus age‐matched males in SNc (*t*_18_ = 2.4, *p* = 0.029) via immunohistochemical labeling of TH and VGLUT2 (Figure 3d). Together, these data demonstrate evolutionarily conserved sex differences in DA neuron VGLUT expression from flies to rodents to humans.

### 2.3 DA neuron dVGLUT knockdown diminishes sex differences in age‐related DA neurodegeneration

We next determined whether there are sex differences in DA neuron survival across aging, and whether loss of dVGLUT expression in DA neurons diminishes such sex differences. For these experiments, the TH promoter drove expression of a GFP marker to selectively label DA neurons in whole living brain preparations from adult flies aged 2, 14, and 60 days post‐eclosion (Figure S2a). We examined several well‐defined DA neuron clusters within the adult central fly brain associated with classic DAergic functions including locomotion and reward processing: the subesophageal ganglion (SOG) cluster, protocerebral anterior lateral (PAL) cluster, and the protocerebral anterior medial (PAM) cluster (Kasture et al., 2018). Using this system, we knocked down dVGLUT expression in DA neurons via previously validated dVGLUT RNA interference (RNAi) (Aguilar et al., 2017). We combined UAS‐GFP and the TH‐GAL4 driver strains via genetic recombination and crossed the progeny to dVGLUT RNAi flies, enabling us to image GFP‐labeled DA neurons where dVGLUT expression has been knocked down. As a control, we confirmed that recombining TH‐GAL4 with GFP does not alter DA neuron numbers across brain regions compared with non‐recombined flies (termed ‘Control flies’; Genotype effect: *F*_1,29_ = 2.1, *p* = 0.16; Bonferroni post hoc test: *p* > 0.16 between genotypes for all regions) (Figure S2b).

**FIGURE 4:**
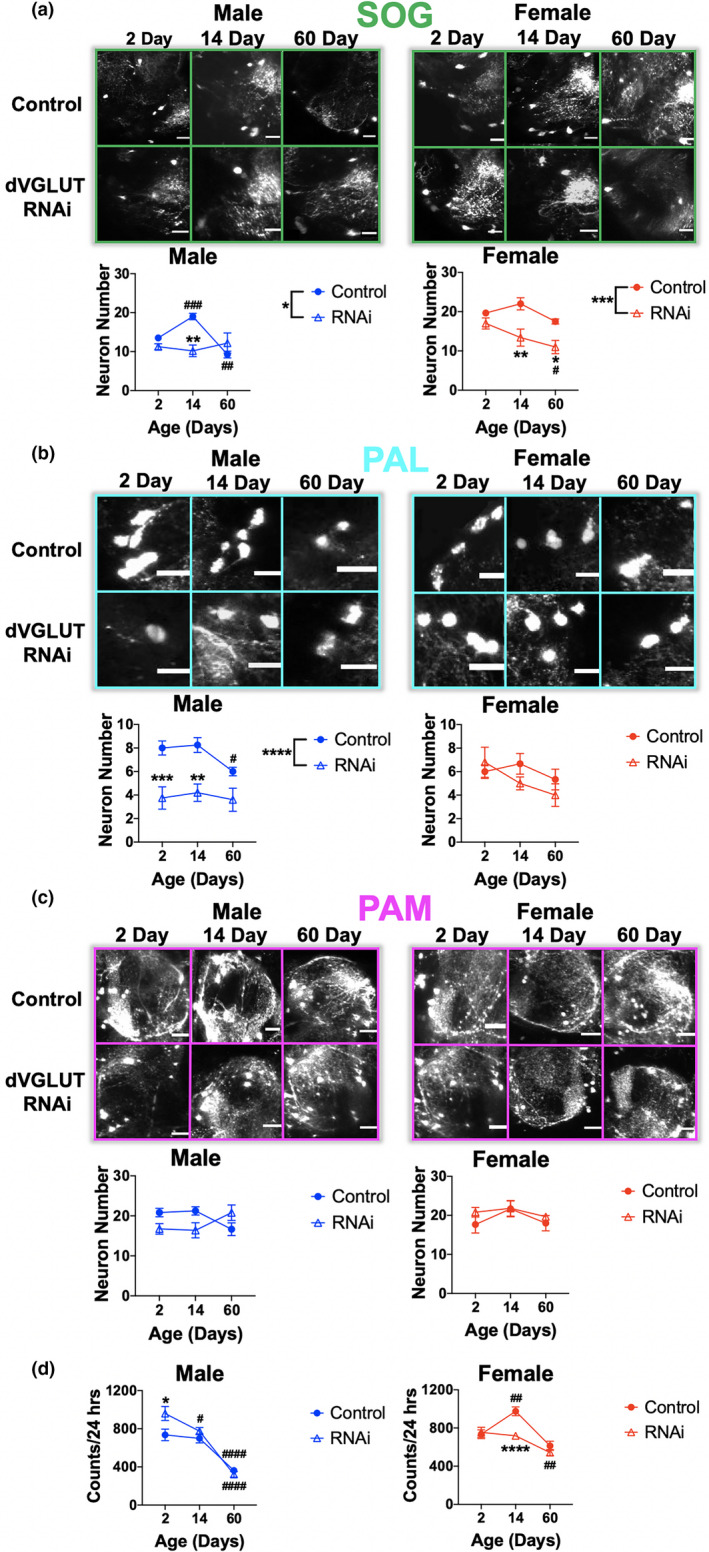
dVGLUT RNAi knockdown diminishes sex‐ and region‐selective DA neuron resilience in aging. (a) Representative multiphoton images of GFP‐labeled DA neurons within the SOG of whole *Drosophila* adult central brain from flies with the following genotypes: TH‐GAL4/UAS‐GFP (Control) (Top Row) versus TH‐GAL4,UAS‐GFP/UAS‐dVGLUT RNAi (dVGLUT RNAi) (Bottom Row); scale bars = 20 μm. (b) Representative multiphoton images of PAL DA neurons; scale bars = 20 μm. (c) Representative multiphoton images of PAM DA neurons; scale bars = 20 μm. (d) Locomotion over a 24‐h period in male and female control and dVGLUT RNAi flies. Results represented as mean ± SEM; *p < 0.05, **p < 0.01, ***p < 0.001, ****p < 0.0001 compared with control by Bonferroni post hoc test, ^#^ *p* < 0.05, ^##^ *p* < 0.01, ^####^ *p* < 0.0001 compared with day 2 post‐eclosion by Bonferroni post hoc test. *n* = 3–8 brains per group for neuron counts and *n* = 24–49 flies for locomotion

We found that dVGLUT knockdown in DA neurons renders SOG DA neurons more vulnerable to age‐related neurodegeneration (Age effect: *F*_2,42_ = 6.0, *p* = 0.0049; dVGLUT RNAi effect: *F*_1,42_ = 22.0, *p* < 0.0001; Age × dVGLUT RNAi interaction: *F*_2,42_ = 5.6, *p* = 0.0068) (Figure 4a). Interestingly, Control males show an increase in GFP‐labeled DA neurons at 14 days compared with 2 days post‐eclosion (Bonferroni post hoc test: *p* = 0.0002). By day 60, however, males have fewer SOG DA neurons compared with day 2 (Bonferroni post hoc test: *p* = 0.0028). Moreover, DA neuron dVGLUT knockdown renders male SOG DA neurons more vulnerable to age‐related DA neurodegeneration, as male dVGLUT RNAi flies demonstrate 50% fewer SOG DA neurons at day 14 post‐eclosion compared with control males (Bonferroni post hoc test: *p* = 0.0011). Critically, whereas Control females show no significant DA neuron loss in the SOG across aging (Bonferroni post hoc test: *p* > 0.99), dVGLUT RNAi females exhibit progressive DA neurodegeneration with 35% of SOG DA neurons lost by day 60 post‐eclosion (Bonferroni post hoc test: *p* = 0.030). In addition, dVGLUT RNAi females have significantly fewer SOG DA neurons compared with controls at 14 days (Bonferroni post hoc test: *p* = 0.0089) and 60 days (Bonferroni post hoc test: *p* = 0.027) post‐eclosion. In the PAL, similar to the SOG, control males show a significant age‐related decrease in PAL DA neurons at day 60 compared with day 2 post‐eclosion (Bonferroni post hoc test: *p* = 0.042), while females are protected with no DA neurodegeneration over time (Bonferroni post hoc test: *p* > 0.99) (Figure 4b). In males, dVGLUT knockdown in DA neurons leads to consistent reduction in PAL DA neurons across timepoints compared with male controls (Sex × dVGLUT RNAi interaction: *F*_1,45_ = 8.0, *p* = 0.0070; Bonferroni post hoc test: Day 2: *p* = 0.0007, Day 14: *p* = 0.0031, Day 60: *p* = 0.068), suggesting a role for dVGLUT in male DA neuron development. In contrast, no significant differences in PAL DA neurons were observed between female dVGLUT RNAi versus control flies (Bonferroni post hoc test: Day 2: *p* > 0.99; Day 14: *p* = 0.82; Day 60: *p* > 0.99). In the PAM, we found no significant effects in males and females (all *F* < 3.0, *p* > 0.07) (Figure 4c), indicating that PAM DA neurons are not significantly impacted by age or sex.

To complement the imaging data, we analyzed locomotion using the same groups and timepoints (Figure 4d). We found that male control flies exhibited age‐related locomotor degeneration while control females did not (Age × Sex interaction: *F*_2,427_ = 12.4, *p* < 0.0001), replicating previous findings (Figure 1). Additionally, dVGLUT RNAi‐mediated knockdown in DA neurons attenuates females' apparent protection from age‐related declines in locomotor behavior, as dVGLUT RNAi females exhibit significant declines in locomotion at day 60 compared with day 2 post‐eclosion (Bonferroni post hoc test: *p* = 0.0070), whereas Control females do not show significant age‐related changes (Bonferroni post hoc test: *p* = 0.21). In contrast to females, Control male dVGLUT RNAi flies demonstrate significant locomotor decreases with aging. However, DA neuron dVGLUT knockdown accelerates these age‐related locomotor declines with diminished locomotion observed at both days 14 and 60 in dVGLUT RNAi flies (Bonferroni post hoc test: Day 2 vs. Day 14: *p* = 0.017; Day 2 vs. Day 60: *p* < 0.0001) compared with only day 60 in Controls (Bonferroni post hoc test: Day 2 vs. Day 14: *p* > 0.99; Day 2 vs. Day 60: *p* < 0.0001). Our results show region‐ and sex‐specific differences in age‐related DA neuron loss. Moreover, considering the age‐related upregulation of DA neuron dVGLUT expression described above, these data point to dVGLUT's importance in both sex differences and age‐mediated increases in DA neuron vulnerability.

### 2.4 Degree of VGLUT2 upregulation determines DA neuron vulnerability versus resilience

Our above findings based on RNAi‐mediated dVGLUT knockdown suggest that endogenous expression of dVGLUT in DA neurons is protective against DA neurodegeneration. By extension, we would predict that enhancing dVGLUT expression in DA neurons would increase resilience to neurodegeneration. Instead, our earlier work demonstrated that ectopic overexpression of dVGLUT in *Drosophila* DA neurons using the strong TH‐GAL4 expression driver led to DA neurodegeneration, rather than offering enhanced neuroprotection (Steinkellner et al., 2018). Significantly, a similar dichotomy was observed in mice. Though VGLUT2‐expressing DA neurons are more resilient to insults and conditional knockout of endogenous VGLUT2 increases these cells' vulnerability to DA neurotoxins (Kouwenhoven et al., 2020; Shen et al., 2018; Steinkellner et al., 2018), Steinkellner et al. (2018) showed that ectopic VGLUT2 overexpression selectively increases DA neuron vulnerability. In contrast, a separate study by Shen et al. (2018) found that VGLUT2 overexpression increases DA neuron resilience to DA neurotoxin 1‐methyl‐4‐phenyl‐1,2,3,6‐tetrahydropyridine (MPTP). These apparent discrepancies led us to probe why knockdown of endogenous VGLUT increased DA neuron vulnerability while overexpression was damaging to DA neurons in one study and protective in another.

**FIGURE 5:**
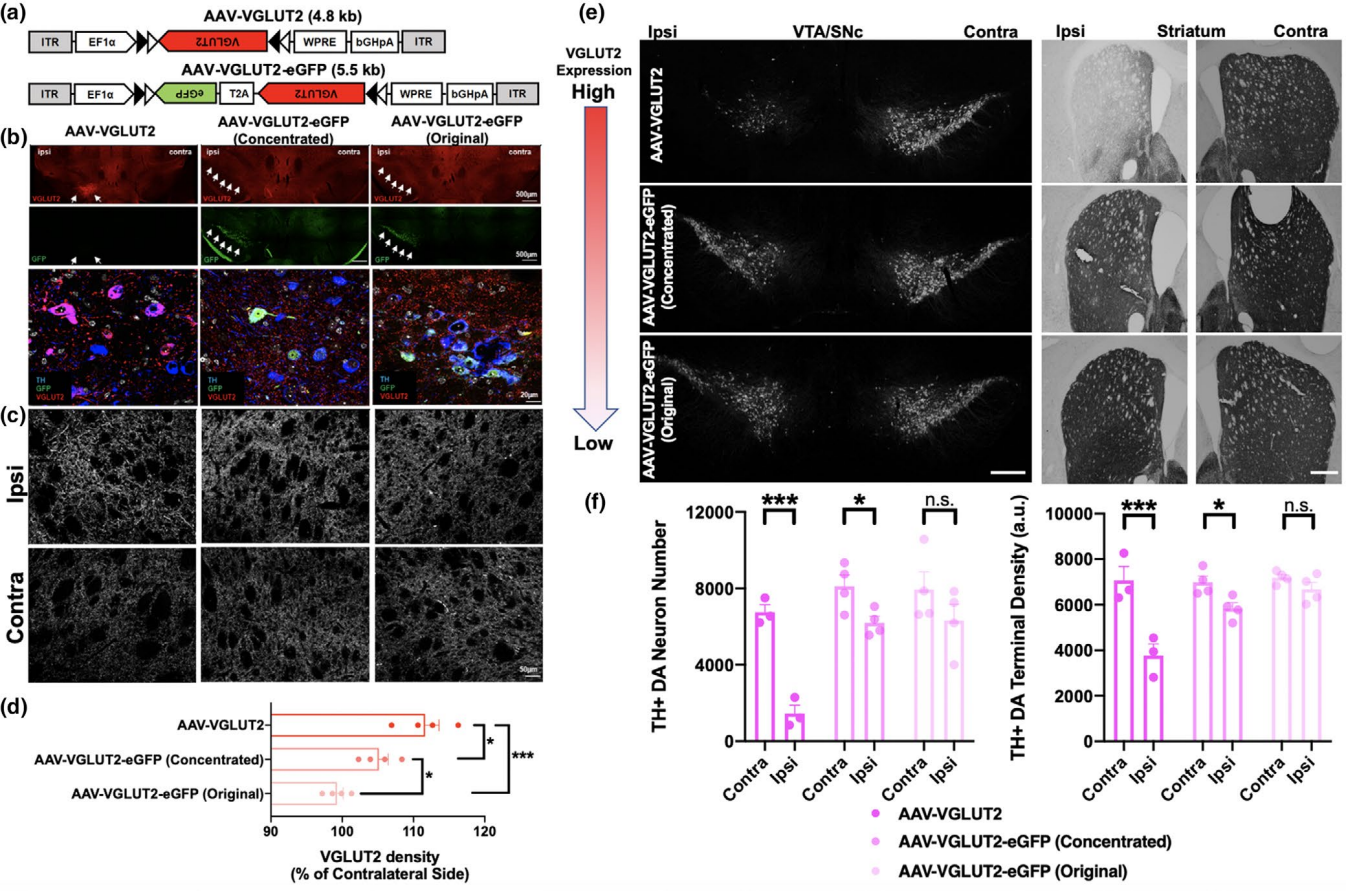
Dopamine (DA) neuron survival in response to different levels of AAV‐mediated VGLUT2 overexpression. (a) Schematic of AAVs used for cell type‐specific VGLUT2 overexpression in SNc DA neurons of DAT^Cre^ mice. (b, c) Detection of VGLUT2 protein in (b) cell bodies, and (c) striatal terminals of SNc DA neurons after unilateral injection of AAV‐VGLUT2 (left panel), AAV‐VGLUT2‐eGFP concentrated titer (middle panel), and AAV‐VGLUT2‐eGFP original titer (right panel) in DAT^Cre^ mice. Ipsi indicates ipsilateral site of viral injection; contralateral side is the uninjected control. (d) Quantification of striatal VGLUT2 density relative to uninjected contralateral sites. (e) Representative midbrain (left panel) and striatal sections (right panel) labeled for TH in mice unilaterally injected with either AAV‐VGLUT2 (upper panel), concentrated titer AAV‐VGLUT2‐eGFP (middle panel), or original titer AAV‐VGLUT2‐eGFP (lower panel); scale bars = 500 μm. (f) Left panel: Quantification of TH^+^ neuron number (left panel) and striatal density of TH^+^ DA nerve terminals (right panel). Results represented as mean ± SEM; *n* = 3–4 animals per group. *p < 0.05, ***p < 0.001 compared by Bonferroni post hoc test

We hypothesized that relative changes to VGLUT expressed in DA neurons may have profound effects on cell survival in the shape of an “inverted U” curve—either too little or too much VGLUT2 expression would increase vulnerability. Such a model could explain the above discrepancies between VGLUT knockdown versus overexpression in DA neurons. We tested our hypothesis in the mouse model, using the AAV system to titrate levels of DA neuron VGLUT2 overexpression. We first examined whether the differences between the two earlier VGLUT2 overexpression studies were related to the respective viruses employed, as the two studies relied on viruses of different genomic sizes and titers. Whereas Steinkellner et al. (2018) used AAV‐VGLUT2 (genome size 4.8 kb, 3.3 × 10^12^ gc/ml), Shen et al. (2018) used AAV‐VGLUT2‐eGFP (genome size 5.5 kb, 2 × 10^13^ gc/ml) (Figure 5a). Thus, to determine whether the two viral preparations produced conflicting effects due to differing levels of DA neuron VGLUT2 overexpression, we unilaterally injected these Cre‐dependent viruses into the SNc of mice expressing Cre recombinase under the control of the dopamine transporter (DAT^Cre^).

Examination of VGLUT2 protein following viral transduction revealed a virus‐dependent gradient of VGLUT2 overexpression in DA neurons and their terminals on the ipsilateral injected side. Highest levels of VGLUT2 overexpression were generated by AAV‐VGLUT2 (111.6 ± 6.2% relative to contralateral side) despite a lower titer (3.3 × 10^12^ gc/ml) compared with AAV‐VGLUT2‐eGFP (2 × 10^13^ gc/ml). Intermediate VGLUT2 overexpression (105.1 ± 4.1% relative to contralateral side) was produced by a titer of AAV‐VGLUT2‐eGFP that was 2.5‐fold more concentrated than originally used by the Shen *et al*. study (5 × 10^13^ gc/ml), with the least overexpression (99.3 ± 2.8% relative to contralateral side) found with the original titer of AAV‐VGLUT2‐eGFP (2 × 10^13^ gc/ml) (Figure 5b,c). While it is important to note that this approach does not discriminate between endogenous (e.g., from thalamic and midbrain inputs) and heterologously expressed VGLUT2, we nevertheless detected a main effect of AAV‐VGLUT2 vector type (*F*_2,9_ = 18.2, *p* = 0.0007) (Figure 5d), suggesting we achieved different levels of DA neuron VGLUT2 overexpression in vivo using different AAVs.

We investigated whether the different levels of VGLUT2 overexpression produced by the AAVs impact DA neuron survival. Transduction with AAV‐VGLUT2 decreased SNc DA neuron number by 79 ± 6.4% (Bonferroni post hoc test: *p* = 0.0001) (Figure 5e,f), similar to our previous report (Steinkellner et al., 2018). In contrast, the original AAV‐VGLUT2‐eGFP did not induce significant DA neuron loss versus the uninjected contralateral side (Bonferroni post hoc test: *p* = 0.058), also consistent with earlier results (Shen et al., 2018). Intermediate levels of VGLUT2 overexpression via the concentrated AAV‐VGLUT2‐eGFP virus reduced TH^+^ DA neuron number by 24 ± 4.1% (Bonferroni post hoc test: *p* = 0.026). Consistent with the respective AAVs' effects on DA neuron number, there was a 47 ± 7.2% loss of TH^+^ DA nerve terminal density in the striatum after AAV‐VGLUT2 vector administration (Bonferroni post hoc test: *p* = 0.00020), compared with 16 ± 3.7% loss for the concentrated AAV‐VGLUT2‐eGFP (Bonferroni post hoc test: *p* = 0.049). Crucially, we observed no significant changes in either DA neuron numbers or terminal density for the original AAV‐VGLUT2‐eGFP titer (Bonferroni post hoc test: DA neuron number: *p* = 0.06, TH terminal density: *p* = 0.65) (Figure 5e,f). Our data therefore suggest that DA neurons require a finely tuned balance of VGLUT2 expression to boost resilience; disrupting this balance either via knockdown or through high levels of overexpression instead increases DA neuron vulnerability to insults.

## 3 DISCUSSION

Our findings demonstrate sex differences in DA neuron vulnerability to age‐related neurodegeneration. Importantly, VGLUT expression is a critical factor for the greater resilience of females to DA cell loss and associated locomotor impairments. Furthermore, we show sex differences in DA neuron VGLUT expression where DA neurons of female flies, rats, and humans express significantly higher levels of VGLUT compared with males. Finally, we demonstrate that the delicate balance of VGLUT2 expression in DA neurons is critical for its effects on resilience.

At the cell level, fly DA neurons are organized into clusters (Mao & Davis, 2009), analogous to the mammalian brain where DA neurons are clustered in the mesencephalon, diencephalon, and olfactory bulbs (Watabe‐Uchida et al., 2012). Moreover, like mammals, fly DA neurons within the clusters project to higher‐order structures (e.g., mushroom bodies and central complex) to modulate behaviors like goal‐directed movement and appetitive motivation (Chen et al., 2013; Riemensperger et al., 2011). Our imaging studies of DA neuron clusters in the anterior protocerebrum including SOG, PAL, and PAM demonstrated age‐dependent DA neuron loss that is region‐specific, preferentially affecting the SOG and PAL clusters while sparing PAM DA cells. Such regional specificity may explain the progressive decline in spontaneous locomotion we observed across aging. Indeed, SOG DA neurons have been implicated in mediating motor control, in addition to their well‐established functions in taste and gustatory reward (Marella et al., 2012). PAL DA neurons are also vulnerable to age‐related DA neurodegeneration based on earlier studies (Bou Dib et al., 2014; Liao et al., 2017). Additionally, our *Drosophila* findings mirror previous reports of region‐specific DA neuron loss in mammals, suggesting that these processes are evolutionarily conserved (Fearnley & Lees, 1991; McGeer et al., 1977). Surprisingly, we also observed a sex‐ and region‐specific increase in DA neuron numbers, finding a rise in DA neuron number within the SOG cluster of adult males between days 2 and 14 post‐eclosion. This raises the possibility that the DA system continues to develop and mature in the adult fly brain. Such a result is consistent with earlier studies showing that DA neuron counts continue to increase in development, rising from the early larval stage to late larval/pupal stage to 3–5 days post‐eclosion in adult flies (Hartenstein et al., 2017). Additionally, previous work showed that whole‐body DA levels rise between 1 and 3 days post‐eclosion, further suggesting ongoing changes within the DA system early in the lives of adult flies, albeit some of these increases may reflect changes in cuticle DA context (Neckameyer et al., 2000). Future studies are therefore needed to definitively delineate these region‐ and sex‐specific changes in the DA system of adult flies as well as the biological mechanisms underlying these dynamic changes during the aging process.

Our imaging studies demonstrated critical sex differences in DA neuron vulnerability across aging. Whereas males exhibit DA neurodegeneration in selected neuron clusters during aging, females are significantly more resilient. These data are consistent with an earlier study in *Drosophila* showing no significant age‐related DA neurodegeneration in females (White et al., 2010). We propose that these sex differences in DA neuron vulnerability may account for the previously reported motor differences between male and female flies (Martin et al., 1999; Niveditha et al., 2017) as well as in mammals (Lacreuse et al., 2005). Nevertheless, the patterns of age‐ and sex‐related effects on motor performance in flies are not entirely consistent across all studies, with genetic background playing a potentially important role in mediating these differences (Fernández et al., 1999; Gargano et al., 2005). Importantly, the sex differences in DA neuron vulnerability described here may also be consistent with preclinical PD models and clinical evidence showing that PD is more prevalent and has an earlier age of onset in men compared with women (Elbaz et al., 2002; Gillies et al., 2014).

This study is the first to investigate VGLUT's role in mediating sex differences in DA neuron vulnerability. We discovered sex differences in DA neuron VGLUT expression in our fly model, finding that females express more DA neuron dVGLUT compared with males. Moreover, these sex differences in VGLUT expression are also present in rodents and humans, suggesting a highly conserved VGLUT‐dependent mechanism underlying these sex‐dependent effects on DA neuron vulnerability. Future studies will examine whether sex hormones including estrogen affect the interactions between sex and DA neuron VGLUT expression and whether this impacts DA neuron vulnerability. Indeed, determining whether manipulating estrogen signaling can alter VGLUT expression to boost DA neuron resilience may create new targets for therapeutic interventions to preserve DA neurons in both healthy aging as well as in PD.

Evidence from our group and others points to a critical role for dynamic regulation of VGLUT expression in determining selective vulnerability of DA neurons (Kouwenhoven et al., 2020; Shen et al., 2018; Steinkellner et al., 2018). Our recent fate‐mapping studies in mice suggest that virtually all DA neurons express VGLUT2 during development, including in the VTA and SNc (Steinkellner et al., 2018). However, by adulthood, most SNc DA neurons no longer express VGLUT2 (Root et al., 2016; Steinkellner et al., 2018). Importantly, we and others also discovered that VGLUT2 expression can reemerge in adult surviving SNc neurons in response to insults that promote neurodegeneration (e.g., 6‐hydroxydopamine) (Kouwenhoven et al., 2020; Steinkellner et al., 2018). Moreover, the proportion of surviving SNc DA neurons that express VGLUT2 is higher compared with the neurons that do not (Steinkellner et al., 2018). This finding may explain our finding of age‐related increases in fly DA neuron dVGLUT expression, despite a decrease in the number of surviving DA neurons at 60 days post‐eclosion in males. Additionally, postmortem brain studies of subjects with known neurodegenerative pathology reported increased VGLUT2 expression in the striatum (Kashani et al., 2007). These findings further support that dynamic changes in DA neuron VGLUT expression may be linked to DA neuron vulnerability in the context of neurodegenerative pathology.

Finally, our work tackles longstanding questions concerning the relationship of VGLUT expression levels to selective DA neuron vulnerability. Our fly data suggest that elevated levels of *endogenous* VGLUT expression boost DA neuron resilience. Disrupting the ability of DA neurons to upregulate VGLUT expression via RNAi‐mediated VGLUT knockdown increases vulnerability to age‐related DA neurodegeneration. These findings are consistent with earlier studies in mice showing that VGLUT2 cKO selectively increases DA neuron vulnerability to DA neurotoxins (Shen et al., 2018; Steinkellner et al., 2018). Conversely, studies that relied on heterologous VGLUT2 overexpression in DA neurons produced conflicting results. In mice, DA neuron‐specific overexpression of VGLUT2 was selectively neuroprotective in one study and neurotoxic in another (Shen et al., 2018; Steinkellner et al., 2018). Similarly, strong dVGLUT overexpression in DA neurons was neurotoxic in adult flies (Steinkellner et al., 2018), suggesting that the mechanisms underlying this phenomenon are conserved across species. In this study, we investigated these conflicting results and discovered that titrating the levels of VGLUT2 overexpression via different AAV viruses and/or viral titers determined whether overexpression altered resilience in midbrain DA neurons. We posit that the AAVs that produce the lowest levels of heterologous VGLUT2 overexpression are likely closest to the levels of endogenous upregulation required to boost DA neuron resilience in response to insults. On the other hand, viruses that generate high physiological levels of VGLUT2 increase selective vulnerability of midbrain DA neurons and their striatal terminals. Since the majority of DA neurons transiently express VGLUT2 during development before repressing its expression in adulthood (Steinkellner et al., 2018), most mature DA neurons may simply not be equipped for sustained, elevated VGLUT2 overexpression. Overall, these data suggest that VGLUT expression in fly and mammalian neurons must be finely tuned through endogenous regulatory mechanisms and that circumventing such well‐conserved mechanisms by altering VGLUT levels at both extremes either through knockdown or via strong ectopic overexpression has a profound effect on cell vulnerability to insults. Future work will define these regulatory mechanisms in DA neurons and their relationships to the sex differences described here.

Taken together, our findings demonstrate sex differences in DA neuron vulnerability to age‐related neurodegeneration. Upregulation of VGLUT in DA neurons with age is neuroprotective and a critical factor for the greater DA neuron and locomotor resilience in females. These results suggest that failures to properly regulate VGLUT expression throughout the lifespan result in heightened DA neuron vulnerability. This suggests a “Goldilocks” model which takes the shape of an inverted “U” curve: Too little VGLUT upregulation may be insufficient to boost neuronal resilience to successfully weather periods of cell stress. When VGLUT2 expression is optimally enhanced in a physiological range, this may compensate for DA system degeneration and enhance DA neuron resilience, as supported by a recent study (Kouwenhoven et al., 2020). On the other hand, either too much VGLUT expression or prolonged duration of overexpression may leave the cells more vulnerable. In such an inverted “U” curve, we also speculate that different subsets of DA neurons and/or different processes within the same cells may be responsible for each arm of the curve. For example, it is possible that an increase in VGLUT2 expression in subsets of DA neurons could have a gain of function effect and increase glutamatergic signaling. This could in turn elevate overall circuit activity and feedback onto the DA neurons to affect their vulnerability. Since DA neuron VGLUT expression could have multiple effects at cell‐ and region‐specific levels, future studies will examine these different possibilities. Ultimately, targeting this VGLUT‐dependent mechanism and its regulation therefore provides opportunities for new therapeutic approaches that can finely tune VGLUT expression to boost DA neuron resilience in both healthy aging and PD.

## 4 EXPERIMENTAL PROCEDURES

### 4.1 *Drosophila* experiments

For *Drosophila* strain construction, drug treatments, and analysis of mRNA expression, see Appendix S1.

#### 4.1.1 *Drosophila* behavior

Locomotor behavior was monitored via the Trikinetics *Drosophila* Activity Monitoring (DAM) system (Trikinetics) as described earlier (Aguilar et al., 2017). Newly eclosed male and female flies were collected daily at fixed, regular times to ensure precise age determination. Adult flies were entrained in 12:12‐h light:dark (LD) cycles at 24°C, ~50% humidity in a humidity‐ and temperature‐controlled incubator. In studies using the wild‐type w^1118^ strain, flies aged 2, 14, 30, or 60 days post‐eclosion were transferred individually into activity tubes containing standard cornmeal‐molasses media and placed into activity monitors (DAM5, Trikinetics); studies examining locomotor behavior of DA neuron‐specific dVGLUT RNAi (*TH*‐*GAL4*,*UAS*‐*GFP*/*UAS*‐*dVGLUT*‐*RNAi*) and control (*TH*‐*GAL4*/*UAS*‐*GFP*) were collected at 2, 14, or 60 days post‐eclosion. Flies were allowed to acclimate to the monitors and individual recording vials for 24 h followed by continuous 24‐h activity monitoring (also in LD) for movement. Locomotor activity was measured as the number of times (counts) a fly crossed the infrared beam running through the middle of each activity tube per 24‐h period. Activity data from the respectively aged flies were averaged and plotted as counts/24 h.

#### 4.1.2 Drug treatments

All drugs were purchased from Sigma‐Aldrich. For luciferase reporter assays, reserpine (300 μM final concentration) was diluted in dimethylsulfoxide and amphetamine (10 mM final concentration) was diluted in distilled water. Drugs and vehicle controls were mixed with molten cornmeal‐molasses media and poured into standard fly vials.

#### 4.1.3 Luciferase reporter assay

Adult DA neuron dVGLUT luciferase reporter flies were transferred into vials either with drug‐ or vehicle‐treated food at 13 days post‐eclosion and dissected 24 h later or dissected at varying ages to analyze age‐related changes in luciferase expression. Fly brains were rapidly dissected from decapitated flies with each sample consisting of five pooled fly brains. Pooled brains included male and female flies unless otherwise specified. Luciferase assays were performed on samples using the Dual‐Luciferase Reporter Assay System (Promega). Brains were homogenized with a pestle in 200 μl of 1x passive lysis buffer. Samples were then shaken at room temperature (45 min, 1200 rpm) and stored at −80°C until the assay was performed (within 1 month). Once thawed, sample lysates were vortexed and diluted in distilled water to ensure that luminescent signal was not oversaturated. Ten μl of each lysate was added into a clear F‐bottom 96‐well plate in triplicate followed by the addition of 100 μl of Luciferase Assay Reagent II to each well in the dark. Luminescence was measured in a Pherastar FSX plate reader (BMG Labtech). Firefly luciferase reactions were terminated with 100 μl of Stop & Glo reagent which also provided an internal control via the *Renilla* luciferase present in the buffer as described earlier (Hirose et al., 2002). Triplicates for each sample were averaged, and results reported as a luminescence ratio of firefly luciferase luminescence divided by *Renilla* luciferase luminescence (LUM ratio).

#### 4.1.4 Multiplex fluorescent in situ hybridization

Multiplex fluorescent in situ hybridization measured mRNA expression as previously described (Long et al., 2017), with adaptations for labeling adult central fly brain. Male and female w^1118^ fly brains were dissected 14 days post‐eclosion in ice‐cold Schneider's insect medium and fixed in 2% paraformaldehyde in PBS. Following wash in PBS with 0.5% Triton X‐100 (PBST), brains were dehydrated in a graded series of ethanol dilutions and shaken in 100% ethanol (4°C, overnight). Brains were then rehydrated with a series of ethanol solutions and incubated with 5% acetic acid to enhance probe penetration. Brains were fixed again in 2% paraformaldehyde in PBS. After washing in PBST, brains were incubated with 1% NaBH_4_ (4°C). After a final PBST wash, brains were transferred onto Superfrost Plus slides (Thermo Fisher Scientific) and air‐dried. Multiplex fluorescent in situ hybridization via RNAscope was performed per manufacturer's instructions (Advanced Cell Diagnostics) to detect mRNA expression of TH (*ple* gene, Cat. No. 536401‐C2) and dVGLUT (*Vglut* gene, Cat. No. 424011). Brains were protease‐treated and probes were hybridized to target mRNAs (2 h, 40°C). After probe amplification, brains were counterstained with DAPI. dVGLUT and TH mRNAs were detected with Alexa 488 and Atto 550, respectively.

#### 4.1.5 Confocal microscopy of *Drosophila* brain fluorescent in situ hybridization

Images were acquired with an Olympus IX81 inverted microscope equipped with a spinning disk confocal unit (Olympus), Hamamatsu EM‐CCD digital camera (Hamamatsu), and BioPrecision2 XYZ motorized stage with linear XYZ encoders (Ludl Electronic Products Ltd) using a 60× 1.4 NA SC oil immersion objective. 3D image stacks (2048 × 2048 pixels, 3 μm in 0.2 μm z‐steps) were taken to ensure full probe penetrance. Image sites were systematically and randomly selected across the fly brain using a grid of 100 μm^2^ frames spaced by 200 μm. Image collection was controlled by Slidebook 6.0 (Intelligent Imaging Innovations, Inc.). Differences in exposures were normalized during image processing.

#### 4.1.6 Ex vivo multiphoton *Drosophila* brain imaging and analysis

Ex vivo whole adult fly brain preparations were obtained by rapid brain removal and microdissection in adult hemolymph‐like saline (AHL, in mM: 108 NaCl, 5 KCl, 2 CaCl_2_, 8.2 MgCl_2_, 1 NaH_2_PO_4_, 10 sucrose, 5 trehalose, 5 HEPES, 4 NaHCO_3_; pH 7.4, 265 mOsm) followed by pinning onto Sylgard slabs with tungsten wire as described previously (Aguilar et al., 2017). The brain preparations were imaged on a Bergamo II resonant‐scanning 2‐photon microscope (Thorlabs Inc.) using a 20× 1.0 NA SemiApo water‐immersion objective (Olympus). The illumination source was an Insight X3 IR laser (Spectra‐Physics, Newport) mode‐locked at 920 nm. Fluorescence emission was collected with a 550/50 nm FWHM bandpass emission filter for GFP (*λ*_ex_ = 920 nm). Z‐stacks through the entire adult central brain were acquired in 2 μm steps using ThorImage 4.0 software (Thorlabs). Images had a voxel size of 0.64 (*x*) × 0.64 (*y*) × 2.00 (*z*) μm with 8× frame averaging. GFP‐labeled cell bodies were counted throughout all z‐stacks from brains of male and female flies ± TH‐driven dVGLUT RNAi at 2, 14, and 60 days post‐eclosion. We focused on DA neuron clusters within the PAL, PAM, and SOG regions (Mao & Davis, 2009). For all cell body counts, comparable results were independently obtained from *n* ≥ 3 blinded experimenters, the median of which was used for analysis. Z‐projections of image stacks were generated for representative images using the Fiji/ImageJ image processing package (National Institutes of Health).

### 4.2 Human brain experiments

#### 4.2.1 Postmortem human subjects

Human postmortem brain specimens (*n* = 2 males, *n* = 2 females) were obtained through the University of Pittsburgh Brain Tissue Donation Program following consent from next‐of‐kin during autopsies conducted at the Allegheny County (Pittsburgh, PA) or Davidson County (Nashville, TN) Office of the Medical Examiner. An independent committee of experienced research clinicians confirmed the absence of lifetime psychiatric and neurologic diagnoses for all subjects on the basis of medical and neuropathological records and structured diagnostic interviews conducted with family members of the deceased (Glausier et al., 2020). Male and female subjects did not differ in mean age, postmortem interval, RNA integrity number, or brain pH (unpaired *t* tests, all *p* > 0.18; Table S1). All procedures were approved by the University of Pittsburgh's Committee for Oversight of Research and Clinical Training Involving Decedents and Institutional Review Board for Biomedical Research.

#### 4.2.2 Fluorescent in situ hybridization

Human fluorescent in situ hybridization was performed using the same protocol as in *Drosophila*, with some modifications for analyzing mRNA in human tissue (see Appendix S1).

### 4.3 Rodent experiments

#### 4.3.1 Animals

Male mice (8–12 weeks old) expressing Cre recombinase under the control of the DA transporter (DAT^cre^) (*Slc6a3^IRESCre^*, Jackson stock 006660, The Jackson Laboratory) were bred on a C57BL/6J genetic background. Mice and adult (10 months) male and female Lewis rats (Envigo) were maintained under standard temperature conditions with a 12:12‐h light:dark cycle; conventional diet and water were available ad libitum. All experiments were approved by the University of Pittsburgh Institutional Animal Care and Use Committee (for rats), and the University of California, San Diego (UCSD) Institutional Animal Care and Use Committee (for mice), which are AAALAC‐accredited. Animals were cared for in accordance with all appropriate animal care guidelines according to the ARRIVE guidelines for reporting animal research. All efforts were made to ameliorate animal suffering. Rodent immunohistochemistry procedures can be found in Appendix S1.

### 4.4 Statistics

Drug‐ and sex‐mediated changes in luciferase reporter expression in flies, sex differences in rat VGLUT2 expression and hemisphere comparisons after AAV‐mediated striatal VGLUT2 overexpression were analyzed via unpaired Student's *t* test. Age‐related changes in luciferase reporter luminescence and striatal VGLUT2 comparisons between mouse viral vectors were analyzed via one‐way analysis of variance (ANOVA). *Drosophila* locomotion and DA neuron numbers in the absence of RNAi‐mediated dVGLUT knockdown were analyzed using two‐way ANOVA, with sex and age as between‐subjects factors. In dVGLUT RNAi flies, locomotion and DA neuron number were analyzed using three‐way ANOVA with sex, age, and dVGLUT RNAi as between‐subjects factors. DA neuron survival and density of striatal DAergic projections after AAV injection were analyzed using repeated measures two‐way ANOVA with AAV preparation as a between‐subjects factor and hemisphere relative to injection as a within‐subjects factor. Significant effects were followed up with Bonferroni post hoc comparisons. In all analyses, statistical significance was defined as *p* < 0.05. All statistical functions were completed using GraphPad Prism (version 8.2; GraphPad Software).

## CONFLICT OF INTEREST

The authors report no competing interests.

## AUTHOR CONTRIBUTIONS

SAB and ZF conceived the project. SAB, DA, MV, SHB, SAR, EIOL, EGN, KJF, MJP, RWL, BDM, CEJC, and ZF performed *Drosophila* experiments with data analysis. BRDM, SAB, and JTG performed rat experiments. TS, SAB, and TSH performed mouse experiments. SAB, JRG, KNF, and DAL performed postmortem human brain experiments. SAB and ZF wrote the manuscript with contributions from co‐authors.

## Supporting information

Appendix S1

Fig S1

Fig S2

## Data Availability

This study includes no data deposited in external repositories. Requests for resources and reagents should be directed to Lead Contact, Zachary Freyberg (freyberg@pitt.edu). Unique reagents generated in this study are available from the Lead Contact with a Materials Transfer Agreement.
