# Supplementary material for: Vesicular glutamate transporter modulates sex differences in dopamine neuron vulnerability to age‐related neurodegeneration
Source: Aging Cell. 2021 Apr 28;20(5):e13365. doi: 10.1111/acel.13365 (PMC8135008; doi:10.1111/acel.13365)
Supplement: Supplementary file 1 — Appendix S1 [file ACEL-20-e13365-s001.docx]

**Supporting Information**

***Drosophila* Strains*.***

All *Drosophila melanogaster* strains were grown and maintained on standard cornmeal-molasses media at 24°C, ~50% humidity under a 12:12 hour light/dark cycle in humidity- and temperature-controlled incubators. Unless otherwise noted, fly stocks were obtained from the Bloomington Stock Center. We used the wild-type w^1118^ strain for locomotor behavioral assays. We also used the following previously described transgenic stocks: *TH-GAL4* (gift of Dr. S. Birman, Université Aix-Marseille II-III, Marseille, France) (Friggi-Grelin et al., 2003) and *TH-LexA* (Berry, Cervantes-Sandoval, Chakraborty, & Davis, 2015) to drive expression in DA neurons via the GAL4/UAS and LexA/LexAop binary expression systems, respectively. *dVGlut-GAL4* was used to drive expression in dVGLUT-expressing neurons (Diao et al., 2015; Sherer et al., 2020). For imaging experiments, to label DA neurons with GFP, *UAS-GFP* (Yeh, Gustafson, & Boulianne, 1995) was genetically recombined with the *TH-GAL4* expression driver on chromosome III to construct the *TH-GAL4,UAS-GFP* fly strain. To ascertain effects of dVGLUT RNAi knockdown on age-related vulnerability of GFP-labeled DA neurons, we crossed *TH-GAL4,UAS-GFP* and *UAS-VGLUT-RNAi* (*UAS-Vglut-RNAi^HMS^*, HMS02011, VALIUM20, target 3077-3098 nt, chromosome 3) (Aguilar et al., 2017; Choi et al., 2014) strains to generate *TH-GAL4,UAS-GFP/UAS-VGLUT-RNAi* flies; as a control, *TH-GAL4,UAS-GFP* flies were crossed to wild-type w^1118^ (*TH-GAL4,UAS-GFP/*+). All fly strains were outcrossed for 10 generations into the w^1118^ wild-type genetic background. For all experiments utilizing drug treatments, flies were randomly assigned to vehicle or drug treatment groups.

**Construction of Transgenic *Drosophila* Strains*.***

*Construction of LexOP>B3R strain*. B3 recombinase was amplified from pJFRC157-20XUAS-IVS-B3::PEST(Nern, Pfeiffer, Svoboda, & Rubin, 2011) (Addgene plasmid #32136) using primers to add a syn21 translational enhancer sequence and remove the PEST domain. The resulting PCR product was transferred into pBID1-13xLexOP, a derivative of the pBID expression vector (Wang, Beck, & McCabe, 2012) containing 13 LexOP sequences. This construct was introduced into the attP40 site on chromosome II by phiC31 injection (Genetivision, Houston, TX).

*Construction of UAS>B3RT-STOP-B3RT-Luciferase* *strain*. The *UAS>B3RT-STOP-B3RT-Luciferase* strain was created by replacement of the myr::RFP sequence in pJFRC160 (Addgene plasmid #32139) (Nern et al., 2011) with firefly luciferase. This modified construct was introduced into the attP2 site on chromosome III by phiC31 injection (Genetivision).

*Construction of the DA neuron dVGLUT luciferase reporter strain*. To measure changes in DA neuron dVGLUT expression, we assembled our intersectional genetic reporter of DA neuron dVGLUT expression using a luciferase reporter: *dVGLUT-GAL4/LexOP>B3R;TH-LexA/UAS>B3RT-STOP-B3RT-Luciferase*. The B3 recombinase recognizes sequence-specific recombination target sites, B3RTs, in a highly specific manner and excises the intervening DNA between recombination sites, leaving behind a single recombinase target site (Nern et al., 2011). Since TH-LexA-driven B3 recombinase is only expressed in DA neurons, this results in cell-specific excision of the B3RT-flanked STOP cassette, enabling dVGLUT-GAL4-driven expression of the intersectional luciferase reporter only in DA neurons.

***Drosophila In Situ* Hybridization Image Analysis and Quantification of mRNA Expression.**

Imaging data was initially analyzed via Slidebook (Intelligent Imaging Innovations, Inc.) and Matlab (MathWorks, Natick, MA) software. First, a Gaussian channel was made for each channel for quantification of mRNA expression by calculating a difference of Gaussians using sigma values of 0.7 and 2. Then, an average projection of each 3D image stack was created by averaging intensity values within each Gaussian channel to assemble a 2D image of DAPI-stained cells and TH and VGLUT2 mRNA transcripts. To quantify mRNA expression within the TH and dVGLUT channels, 2D projection images were separated into quantitative TIFF files of each individual Gaussian channel and transferred to the HALO image analysis platform equipped with a fluorescent *in situ* hybridization add-on (Version 1.7, Indica Labs, Albuquerque, NM). DAPI-stained cell nuclei and fluorescent grains representing mRNA transcripts from the VGLUT2 and TH channels were quantified via HALO software using the following parameters for inclusion in our counts based on the following thresholding criteria: any object 1-10μm^2^ for DAPI and 0.03-0.15μm^2^ for TH and dVGLUT grains. We quantified the respective expression levels of TH and dVGLUT mRNA in positive cells as described earlier (Rocco, Sweet, Lewis, & Fish, 2016), with some modifications. To determine the minimum number of mRNA grains associated with a DAPI-stained nucleus considered positive, we tested different thresholds (TH: 50-, 100-, and 200-times; dVGLUT: 10-, 25-, and 50-times) of the number of mRNA grains above background levels (*i.e.*, the number of grains expressed in a typical cell volume). Since there were no significant differences in relative TH^+^/dVGLUT^+^ cell densities between the thresholds (unpaired t-tests; all p>0.11), minimum thresholds of 100-times the background expression level for TH and 25-times the background expression level for dVGLUT were selected as the thresholds for quantifying positive cells. All mRNA grains within 1μm of the nucleus edge were considered to belong to the respective cell, and this 1μm border was reduced in HALO whenever necessary to prevent overlap between neighboring cells. TH and dVGLUT cell expression were quantified together and reported as density of TH^+^/dVGLUT^+^ cells. Experimenters were blinded to sex of subjects until analysis was completed.

**Human Fluorescent *In Situ* Hybridization**

*In situ* hybridization probes for multiplex fluorescent *in situ* hybridization in human brain samples were designed by Advanced Cell Diagnostics to detect mRNAs encoding TH (*TH* gene, Cat. No. 441651-C2) and VGLUT2 (*SLC17A6* gene, Cat. No. 415671). Right hemisphere midbrain containing the VTA and SNc were cut at a 20μm thickness using a cryostat, mounted onto Superfrost Plus slides (Thermo Fisher Scientific) and stored at -80°C until processing. Tissue sections (2 per subject) were processed within a month of sectioning, and one section from each subject was processed on the same day to reduce batch effects. Multiplex fluorescent *in situ* hybridization via RNAscope was performed according to manufacturer’s instructions (Advanced Cell Diagnostics). Briefly, tissue sections were fixed for 15 min in ice-cold 4% paraformaldehyde, incubated in a protease treatment, and then the probes were hybridized to their target mRNAs (2 hours, 40°C). The sections were exposed to a series of incubations that amplified the target probes, and then counterstained with DAPI. VGLUT2 and TH mRNAs were detected with Alexa 488 and Atto 550, respectively.

**Confocal Microscopy Imaging of Human Brain Sections Processed for Fluorescent *In Situ* Hybridization.**

Images were acquired with an Olympus IX81 inverted microscope equipped with an Olympus spinning disk confocal unit, a Hamamatsu EM-CCD digital camera and a high-precision BioPrecision2 XYZ motorized stage with linear XYZ encoders (Ludl Electronic Products Ltd) using a 60x 1.4 NA SC oil immersion objective. 3D image stacks (2048×2048 pixels; 0.2μm z-steps) of 100% of the tissue thickness were taken in the VTA and SNc spanning the entire medial-lateral and dorsal-ventral axes. Image sites were systematically and randomly selected using a grid of 100μm^2^ frames spaced by 350μm. Image collection was controlled by Slidebook 6.0 (Intelligent Imaging Innovations, Inc.). The z-stacks were collected using optimal exposure settings (*i.e.*, those that yielded the greatest dynamic range with no saturated pixels), with differences in exposures normalized during image processing. Lipofuscin, an intracellular lysosomal protein that accumulates with age (Benavides, Monserrat, Farina, & Porta, 2002; Porta, Berra, Monserrat, & Benavides, 2002), is a major source of native fluorescence across the visible spectrum in human postmortem tissue; however, we effectively exclude lipofuscin signal from our RNAscope probe-specific signals in human samples using a previously described approach (Curley et al., 2011; Fish, Rocco, & Lewis, 2018; Glausier, Fish, & Lewis, 2014; Rocco, DeDionisio, Lewis, & Fish, 2017; Rocco, Lewis, & Fish, 2016; Sweet, Fish, & Lewis, 2010). Specifically, we imaged lipofuscin using a fourth visible channel (excitation/emission: 405 nm/647 nm) and masked the lipofuscin signal using an optimal threshold value.

**Image Analysis and Quantification of mRNA Expression in Human Brain.**

Imaging data was initially analyzed via Slidebook (Intelligent Imaging Innovations, Inc.) and Matlab software (MathWorks). A Gaussian channel was made for each channel by calculating a difference of Gaussians using sigma values of 0.7 and 2. Average projections of each 3D image stack were subsequently created by averaging intensity values within each Gaussian channel to assemble a 2D image of DAPI-stained cells and TH and VGLUT2 mRNA transcripts. Objects from the other channels that overlapped with lipofuscin were eliminated from analyses by subtracting the lipofuscin Gaussian channel from the other channels. To quantify mRNA expression within the TH and VGLUT2 channels, 2D projection images were separated into quantitative TIFF files of each individual Gaussian channel and transferred to the HALO image analysis platform equipped with a fluorescent *in situ* hybridization add-on (Version 1.7, Indica Labs). DAPI-stained cell nuclei and fluorescent grains representing mRNA transcripts from the VGLUT2 and TH channels were quantified using the following inclusion parameters based on our thresholding: any object 40-500μm^2^ for DAPI and 0.1-0.5μm^2^ for TH and VGLUT2. Positive cell expression levels were determined as previously described (Rocco, Sweet, et al., 2016). To determine the minimum number of mRNA grains associated with a DAPI-stained nucleus considered positive, we tested different thresholds of 1.5-, 3- and 5-times the number of mRNA grains above background levels. Because there were no significant differences in relative TH^+^/VGLUT2^+^ cell densities between the thresholds (unpaired t-tests; all p>0.36), a minimum threshold of 3-times the background expression level was chosen as the threshold for quantifying positive cells. All mRNA grains within 5μm of the nucleus edge were considered as belonging to the respective cell, and this 5μm border was reduced in HALO whenever necessary to prevent overlap between neighboring cells. TH and VGLUT2 mRNA expression in neurons within the VTA and SNc were quantified together and reported as density of TH^+^/VGLUT2^+^ cells. Experimenters were blinded to sex of subjects until analysis was completed.

**Rat Immunohistochemistry and Image Analysis.**

Rats were euthanized using pentobarbital, followed by transcardial perfusion and 4% paraformaldehyde fixation. Brains were fixed in paraformaldehyde for 24-hours and transferred to 30% sucrose at 4°C until sectioning. Nigral sections (35μm) were sliced on a freezing microtome and maintained by free-floating in cryoprotectant at -20°C until immunohistochemical labeling. Sections were labeled for TH (1:2000, AB1542, EMD Millipore, Burlington, MA) and VGLUT2 (1:500, 135403, Synaptic Systems, Goettingen, Germany), then mounted onto glass slides for imaging using a “primary antibody delete” (secondary antibody only) stained section to subtract background fluorescence as described earlier(De Miranda, Fazzari, Rocha, Castro, & Greenamyre, 2019). Images were acquired using an Olympus BX61 microscope and Fluoview 1000 software (Nikon, Melville, NY). Quantitative fluorescence measurements were monitored using standard operating imaging parameters to ensure the absence of saturated pixels during image acquisition. For quantitative comparisons, all imaging parameters (*e.g.*, laser power, exposure, and pinhole) were held constant across specimens. At least 6 images were analyzed per animal with analysis performed using Nikon NIS-Elements Advanced Research software (Version 4.5; Nikon, Melville, NY). Results are reported as a count of VGLUT2 puncta within TH^+^ cells (# objects/TH^+^ Cell).

**Mouse Viruses.**

We used serotype 1, replication-incompetent, Adeno-associated viruses (AAV) to drive expression of VGLUT2 under the control of the EF1α promoter: AAV1-EF1α-DIO-VGLUT2 (referred to as AAV-VGLUT2 in results) as described previously (Steinkellner et al., 2018). The virus was packaged at the Salk GT3 vector core (La Jolla, CA). We also used serotype 5 AAV5-EF1α-DIO-VGLUT2-T2A-eGFP (referred to as AAV-VGLUT2-eGFP in results) as reported earlier(Shen et al., 2018); this virus was packaged by Vigene Biosciences (Rockville, MD).

**Mouse Viral injections.**

Mice were anaesthetized with isoflurane (2-5%) and placed into a stereotaxic frame (David Kopf Instruments, Tujunga, CA) and randomly assigned one of three viruses. 300 nl of the following viruses were used: AAV-VGLUT2 (3.3×10^12^ genome copies per ml [gc/ml]), AAV-VGLUT2-eGFP (5×10^13^ gc/ml; concentrated titer), or PBS-diluted AAV-VGLUT2-eGFP (2×10^13^ gc/ml; originally reported titer (Shen et al., 2018)). The respective viruses were microinfused into the left SNc (-3.4 anterior-posterior, -1.25 medial-lateral, -4.25 dorsal-ventral; in millimeters from bregma) using custom-made 30G stainless steel injectors at a speed of 100nl/min.

**Mouse Immunohistochemistry.**

Mice were deeply anaesthetized ﻿with pentobarbital (200 mg/kg i.p.; Virbac Corp., Westlake, TX) and transcardially perfused with 10–20ml of phosphate-buffered saline (PBS) 3 weeks after viral injection. This was followed by perfusion with 60-70ml of 4% paraformaldehyde (PFA) at a rate of 6 ml/min. Brains were extracted, post-fixed in 4% PFA at 4°C overnight, and cryoprotected in 30% sucrose in PBS for 48–72 hours at 4°C. Brains were snap-frozen in chilled isopentane and stored at -80°C. Sections (30μm) were sectioned using a cryostat (CM3050S, Leica, Wetzlar, Germany) and collected in PBS containing 0.01% sodium azide.

For fluorescent immunostaining, brain sections were blocked with 5% normal donkey serum in PBS containing 0.3% Triton X-100 (blocking buffer) (1-hour, room temperature). Sections were then incubated with one or more of the following primary antibodies (rabbit anti-TH, 1:2000, AB152, EMD Millipore; guinea pig anti-VGLUT2, AB2251, EMD Millipore; chicken anti-GFP, A10262, Invitrogen, Carlsbad, CA) in blocking buffer (overnight, 4°C). Sections were rinsed 3×15 min with PBS and incubated in appropriate secondary antibodies (Jackson ImmunoResearch, West Grove, PA) conjugated to Alexa 488, Alexa 594 or Alexa 647 fluorescent dyes (5 μg/ml) (2-hours, room temperature). Sections were washed 3×15 min with PBS, mounted onto glass slides and coverslipped with Fluoromount-G mounting medium (Southern Biotech, Birmingham, AL) supplemented with DAPI stain (0.5 µg/ml, Roche, Basel, Switzerland). Images were acquired using a Zeiss AxioObserver epifluorescence microscope (Oberkochen, Germany).

For TH-DAB staining, sections were quenched in 3% H_2_O_2_ in PBS (30 min, room temperature) before blocking (1-hour, room temperature). Sections were incubated with rabbit anti-TH primary antibody (1:2000; AB152, Millipore) in blocking buffer (overnight, 4°C). The following day, sections were washed 3×15 min with PBS and incubated with a donkey anti-rabbit biotinylated secondary antibody (Jackson ImmunoResearch) at 1:500 in blocking buffer (2-hours, room temperature). Sections were again washed 3×15 min with PBS and incubated in avidin-biotin complex solution (Vectastain Elite ABC kit, Vector Laboratories, Burlingame, CA) (2-hours, room temperature) before 2 additional PBS washes (10 minutes). Sections were incubated in DAB solution (0.4mg/ml 3,3-diaminobenzidine-HCl, 0.005% H_2_O_2_ in PBS) (3 min, room temperature). Sections were rinsed twice in PBS before mounting onto glass slides and drying overnight. Sections were then dehydrated through increasing concentrations of ethanol and isopropanol, cleared with CitriSolv (Thermo Fisher Scientific, Waltham, MA), and cover-slipped using DPX mounting medium (Sigma-Aldrich, St. Louis, MO).

**Unbiased Stereology.**

Stereological sampling was performed using the Stereo Investigator (SI) software (MBF Bioscience, Williston, VT). Counting frames (100×100µm) were randomly placed on a counting grid (200×200 µm) and sampled using a 7-µm optical dissector with guard zones of 10% of the total slice thickness on each site (~2 µm). The boundaries of SNc were outlined under magnification (4x objective). Cells were counted with a 20x objective using a Zeiss AxioImager microscope (Carl Zeiss Microscopy, White Plains, NY). A DAergic neuron was defined as an in-focus TH-DAB-immunoreactive (TH-IR) cell body with a TH-negative nucleus within the counting frame. Every sixth section was processed for TH-IR, resulting in 6-7 sections containing SNc sampled per mouse and every section was counted. The number of neurons in SNc was estimated using the optical fractionator method, which is unaffected by changes in the volume of reference of the structure sampled.

**TH and VGLUT2 Densitometry.**

Images (TH-DAB stained) were acquired using a Zeiss AxioObserver microscope (Carl Zeiss Microscopy) under brightfield illumination. Four striatal sections per animal were analyzed using ImageJ software (National Institutes of Health). Regions of interest in the dorsal striatum were delineated, pixel densities were estimated and intensities over the four sections were averaged.

**Sample Size Estimation**

G*Power was used to estimate sample sizes (Faul, Erdfelder, Lang, & Buchner, 2007) using a power of 0.80 and α of 0.05. For locomotion experiments, which were compared via three- or two-way ANOVAs, a small estimated effect size (partial η^2^) of 0.03 resulted in a predicted sample size of 38 flies per group. For the dVGLUT RNAi locomotion experiment, 25-49 flies per group were used, and a power of 0.96 was achieved. Therefore, for the wild-type locomotion experiment, less (11-31) flies were used per group, and a power of 0.86 was achieved. Luciferase experiments, which were compared by unpaired t-tests, were expected to have low variability (due to 5 homogenized brains used per n) and large effect sizes. This led to a predicted effect size (Cohen’s d) of 4, which resulted in a predicted minimum sample size of 3 per group. For luciferase experiments compared by one-way ANOVA, a large estimated effect size (partial η^2^) of 0.3 resulted in a predicted sample size of 7 per group. For experiments on sex differences in DA neuron VGLUT expression in flies and humans (compared by unpaired t-tests), a large predicted effect size (Cohen’s d) of 4 resulted in a predicted minimum sample size of 3 per group. A smaller effect size (Cohen’s d) of 1.25 was expected in measurement of VGLUT2 puncta in DA neurons in rats, resulting in a predicted sample size of 9 per group. The *Drosophila* DA neuron counts compared by three-way ANOVA had a predicted large effect size of (partial η^2^) of 0.2, resulting in a predicted sample size of 5 per group. Finally, the AAV experiments that were compared by one- and two-way ANOVAs had a large predicted effect size (partial η^2^) of 0.3 which resulted in a predicted sample size of 4 per group.

**Figure S1 - Original intersectional DA neuron dVGLUT luciferase reporter luminescence and controls.** Female flies express 2-fold more dVGLUT in DA neurons compared to males. The TH-LexA and dVGLUT-GAL4-driven intersectional genetic reporter of DA neuron dVGLUT expression expresses 1200-fold more luminescence compared to the undriven (LexAop-B3R/+;UAS-B3RT.STOP.B3RT.Luciferase/+) or wild-type w^1118^ controls of either sex. There are no significant differences in firefly:*Renilla* luminescence ratios between the controls (one-way ANOVA: p=0.36). Results represented by mean±SEM; *p<0.05 by unpaired t-test, n=3-4 homogenates of 5 brains per group.

| **Subject** | **Sex** | **Age (years)** | **Postmortem interval (hours)** | **Brain pH** | **RNA Integrity Number** |
| --- | --- | --- | --- | --- | --- |
| 1 | Male | 17 | 15.1 | 6.9 | 7.9 |
| 2 | Male | 22 | 20.1 | 6.9 | 7.8 |
| 3 | Female | 16 | 9.3 | 6.6 | 9.0 |
| 4 | Female | 21 | 23.9 | 6.8 | 8.2 |

**Table S1 -** Characteristics of human subjects. Male and female subjects were matched with no significant differences in mean age, postmortem interval, brain pH or RNA integrity number (two-tailed unpaired t-test: all p>0.05).

**Figure S2 - GFP-labeled DA neurons in whole intact male and female *Drosophila* brains, and comparison of flies with and without recombination of TH-GAL4 and UAS-GFP**. (a) Representative two-dimensional (2D) projection images of multiphoton microscopy of living whole fly brains of male and female adult flies (TH-GAL4/UAS-GFP) aged 2- versus 60-days post-eclosion. Insets highlight SOG (green box), PAL (white box), and PAM (magenta box) DA neuron clusters and show age-related loss of PAL and SOG DA neurons in males, but not in females. Scale bars of 2D whole brain projections=100μm, scale bars of insets=20μm. (b) DA neurons were counted in DA neuron clusters in the PAL, PAM and SOG brain regions imaged by multiphoton microscopy of whole living *Drosophila* brains of 2-day-old adult males. DA neurons were labeled by combining the TH-GAL4 expression driver and UAS-GFP via genetic recombination (TH-GAL4, UAS-GFP/+; termed ‘Recombined’). The DA neurons in brains of Recombined flies were compared to GFP-labeled DA neurons in unrecombined flies (TH-GAL4/UAS-GFP; termed ‘Unrecombined’; Bonferroni post-hoc test: p>0.16 between genotypes for all regions). Results represented as mean±SEM; n=4-8 brains per group.

**Supporting Information References**

Aguilar, J. I., Dunn, M., Mingote, S., Karam, C. S., Farino, Z. J., Sonders, M. S., . . . Freyberg, Z. (2017). Neuronal Depolarization Drives Increased Dopamine Synaptic Vesicle Loading via VGLUT. *Neuron, 95*(5), 1074-1088 e1077. doi:10.1016/j.neuron.2017.07.038

Berry, J. A., Cervantes-Sandoval, I., Chakraborty, M., & Davis, R. L. (2015). Sleep Facilitates Memory by Blocking Dopamine Neuron-Mediated Forgetting. *Cell, 161*(7), 1656-1667. doi:10.1016/j.cell.2015.05.027

Choi, B. J., Imlach, W. L., Jiao, W., Wolfram, V., Wu, Y., Grbic, M., . . . McCabe, B. D. (2014). Miniature neurotransmission regulates Drosophila synaptic structural maturation. *Neuron, 82*(3), 618-634. doi:10.1016/j.neuron.2014.03.012

Diao, F., Ironfield, H., Luan, H., Diao, F., Shropshire, W. C., Ewer, J., . . . White, B. H. (2015). Plug-and-play genetic access to drosophila cell types using exchangeable exon cassettes. *Cell Rep, 10*(8), 1410-1421. doi:10.1016/j.celrep.2015.01.059

Faul, F., Erdfelder, E., Lang, A. G., & Buchner, A. (2007). G*Power 3: a flexible statistical power analysis program for the social, behavioral, and biomedical sciences. *Behav Res Methods, 39*(2), 175-191. doi:10.3758/bf03193146

Friggi-Grelin, F., Coulom, H., Meller, M., Gomez, D., Hirsh, J., & Birman, S. (2003). Targeted gene expression in Drosophila dopaminergic cells using regulatory sequences from tyrosine hydroxylase. *J Neurobiol, 54*(4), 618-627. doi:10.1002/neu.10185

Nern, A., Pfeiffer, B. D., Svoboda, K., & Rubin, G. M. (2011). Multiple new site-specific recombinases for use in manipulating animal genomes. *Proc Natl Acad Sci U S A, 108*(34), 14198-14203. doi:10.1073/pnas.1111704108

Sherer, L. M., Catudio Garrett, E., Morgan, H. R., Brewer, E. D., Sirrs, L. A., Shearin, H. K., . . . Certel, S. J. (2020). Octopamine neuron dependent aggression requires dVGLUT from dual-transmitting neurons. *PLoS Genet, 16*(2), e1008609. doi:10.1371/journal.pgen.1008609

Wang, J. W., Beck, E. S., & McCabe, B. D. (2012). A modular toolset for recombination transgenesis and neurogenetic analysis of Drosophila. *PLoS One, 7*(7), e42102. doi:10.1371/journal.pone.0042102

Yeh, E., Gustafson, K., & Boulianne, G. L. (1995). Green fluorescent protein as a vital marker and reporter of gene expression in Drosophila. *Proc Natl Acad Sci U S A, 92*(15), 7036-7040. doi:10.1073/pnas.92.15.7036
