# Supplementary material for: Vesicular glutamate transporter modulates sex differences in dopamine neuron vulnerability to age‐related neurodegeneration
Source: Aging Cell. 2021 Apr 28;20(5):e13365. doi: 10.1111/acel.13365 (PMC8135008; doi:10.1111/acel.13365)
Supplement: Supplementary file 2 — Fig S1 [file ACEL-20-e13365-s003.pdf]

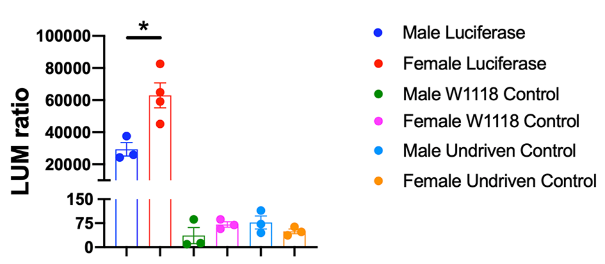

*
LUM ratio
100000
80000
60000
40000
20000
150
75
0
Male Luciferase
Female Luciferase
Male W1118 Control
Female W1118 Control
Male Undriven Control
Female Undriven Control
