# Supplementary figures and images for: Vesicular glutamate transporter modulates sex differences in dopamine neuron vulnerability to age‐related neurodegeneration
Source: Aging Cell. 2021 Apr 28;20(5):e13365. doi: 10.1111/acel.13365 (PMC8135008; doi:10.1111/acel.13365)

**a****Male****Female****2 Days**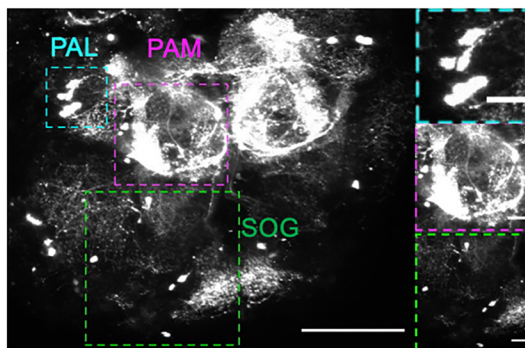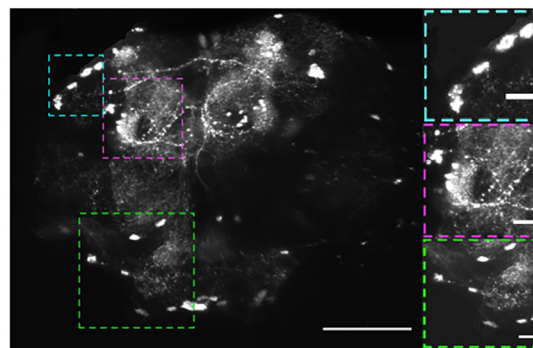**60 Days**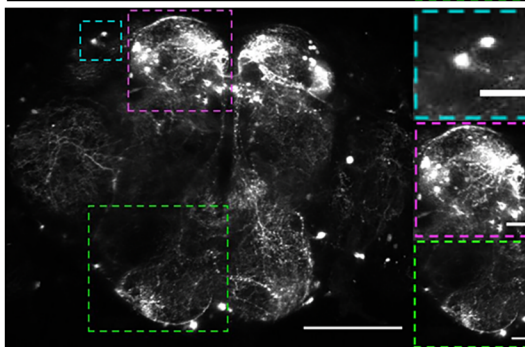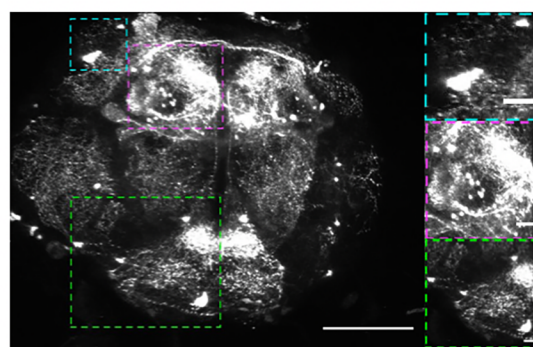**b**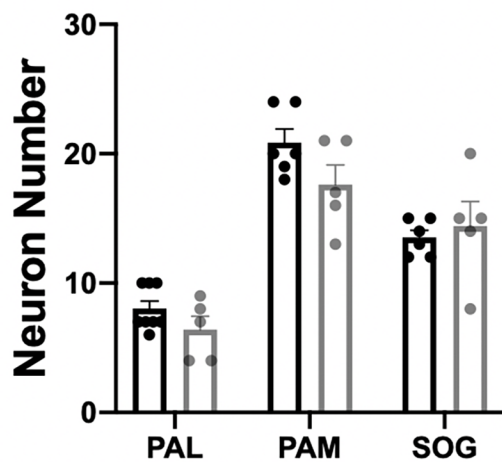

- TH-GAL4/UAS-GFP (Unrecombined)
- TH-GAL4, UAS-GFP/+ (Recombined)

Supplement: Supplementary file 3 — Fig S2 [file ACEL-20-e13365-s002.pdf]
